# Extreme adaptations for probable visual courtship behaviour in a Cretaceous dancing damselfly

**DOI:** 10.1038/srep44932

**Published:** 2017-03-20

**Authors:** Daran Zheng, André Nel, Edmund A. Jarzembowski, Su-Chin Chang, Haichun Zhang, Fangyuan Xia, Haoying Liu, Bo Wang

**Affiliations:** 1 State Key Laboratory of Palaeobiology and Stratigraphy, Nanjing Institute of Geology and Palaeontology, Chinese Academy of Sciences, 39 East Beijing Road, Nanjing 210008, China; 2 Department of Earth Sciences, The University of Hong Kong, Hong Kong Special Administrative Region, China; 3 Institut de Systématique, Évolution, Biodiversité, ISYEB-UMR 7205-CNRS, MNHN, UPMC, EPHE, Muséum national d’Histoire naturelle, Sorbonne Universités, 57 rue Cuvier, CP 50, Entomologie, F-75005, Paris, France; 4 Department of Earth Sciences, The Natural History Museum, London SW7 5BD, UK; 5 Nanjiao Bieshu 394, Shanghai, China; 6 Key Laboratory of Zoological Systematics and Evolution, Institute of Zoology, Chinese Academy of Sciences, 1, Beichen West Road, Beijing 100101, China

## Abstract

Courtship behaviours, frequent among modern insects, have left extremely rare fossil traces. None are known previously for fossil odonatans. Fossil traces of such behaviours are better known among the vertebrates, e.g. the hypertelic antlers of the Pleistocene giant deer *Megaloceros giganteus*. Here we describe spectacular extremely expanded, pod-like tibiae in males of a platycnemidid damselfly from mid-Cretaceous Burmese amber. Such structures in modern damselflies, help to fend off other suitors as well as attract mating females, increasing the chances of successful mating. Modern Platycnemidinae and Chlorocyphidae convergently acquired similar but less developed structures. The new findings provide suggestive evidence of damselfly courtship behaviour as far back as the mid-Cretaceous. These data show an unexpected morphological disparity in dancing damselfly leg structure, and shed new light on mechanisms of sexual selection involving intra- and intersex reproductive competition during the Cretaceous.

Courtship behaviour is quite frequent among in extant insects^1^,^2^. In odonates, the male must persuade the female to mate in tandem and the female should be willing to engage her genitalia with the male’s^3^. But in some situations, the male calopterygid damselfly may force the female copulations^4^. Many territorial odonatans display their courtship by high-frequency wing-beats towards an approaching female^5^,^6^. Most courtship, mating and parenting (social-sexual) behaviour cannot be preserved and fossil reports are few and ambiguous^7^. Some cases of insect mating behaviour are better preserved in amber compared with rare records in sedimentary rocks^8^,^9^,^10^. Also brood care behaviour has been recorded in fossil insects^11^,^12^,^13^. However, direct evidence of courtship behaviour in fossils is extremely rare: male mecopterans have exaggerated body parts used for sexual display^14^ and the male *Karataus* Rasnitsyn^15^ (Middle Jurassic, Hymenoptera) has swollen hind femora used for courtship display or to assist attachment to the female^15^,^16^. The male Strashilidae (Middle Jurassic, Diptera) have swollen hind tibiae and femora probably for a similar function^17^. The bug *Gyaclavator kohlsi* (Eocene, Heteroptera) has the antennae with dilated distiflagellomere probably used for competition and attraction behavior^18^. The absence of a tangible fossil record, however, limits our understanding of the origin and evolution of courtship behaviour in dragonflies^19^.

Here we describe a new damselfly with expanded tibiae uniquely probably used for courtship from mid-Cretaceous Burmese amber. The damselfly is attributed to the recent family Platycnemididae. The specimens described herein were collected in the Hukawng Valley of Kachin Province, Myanmar (locality in Kania *et al*.^20^: Fig. 1). The age of the Burmese amber matrix was radiometrically dated at 98.79 ± 0.62 Ma (earliest Cenomanian) based on U–Pb zircon dating of the volcanoclastic matrix^21^. The insect inclusions in Burmese amber have been studied for about a century^20^,^21^,^22^, but only a few odonatans have been recorded, all in the present decade^23^,^24^,^25^,^26^,^27^,^28^,^29^,^30^,^31^. The new find reveals ancient courtship, insect interaction and sexual selection from as far back as the mid-Cretaceous.

## Results

### Systematic palaeontology

Order Odonata Fabricius, 1793

Suborder Zygoptera Selys-Longchamps, 1854

Superfamily Coenagrionoidea Kirby, 1890

Family Platycnemididae Yakobson & Bianchi, 1905

Subfamily Palaeodisparoneurinae Poinar *et al*.^9^

### Yijenplatycnemis huangi gen. et sp. nov

**Figure 1:**
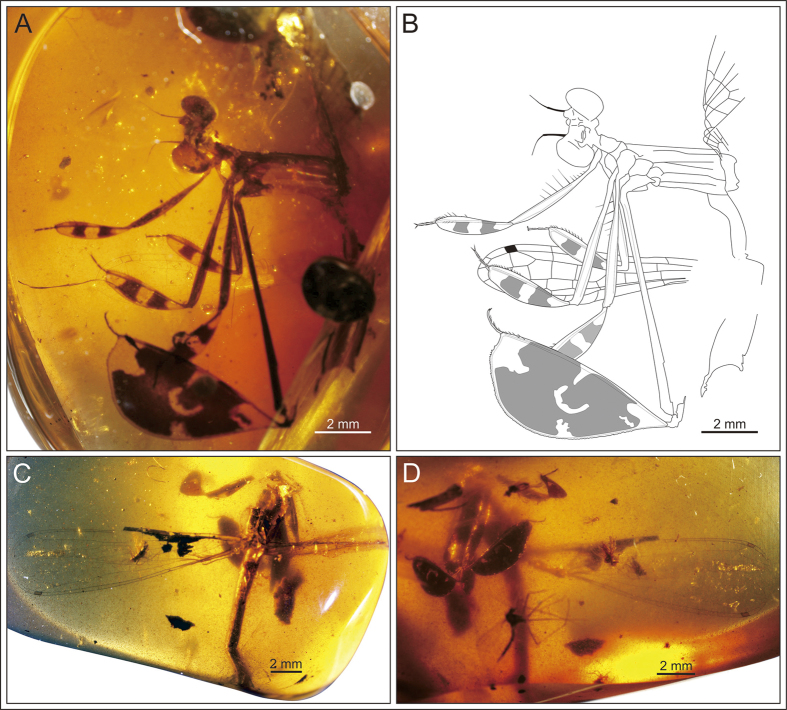
*Yijenplatycnemis huangi* gen. et sp. nov. Holotype (NIGP164757); photograph (**A**) and line drawing (**B**) of specimen (drawn by DZ). Paratype (BA16200); dorsal view (**C**) and anterior view (**D**) of specimen.

**Figure 2:**
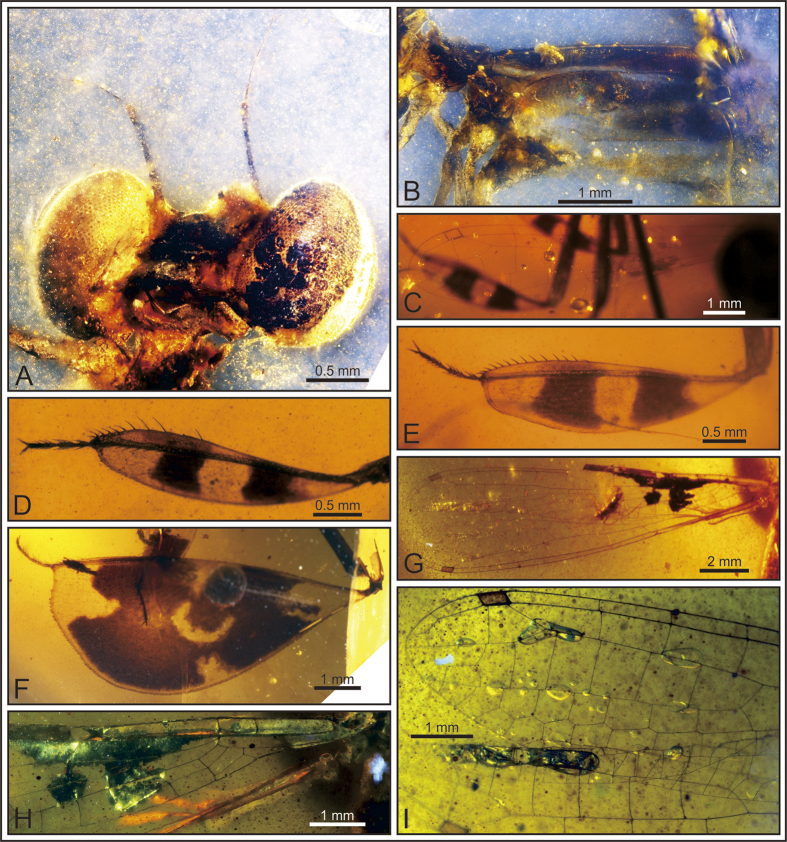
*Yijenplatycnemis huangi* gen. et sp. nov. (**A–F**) Holotype (NIGP164757); (**G–I**) paratype (BA16200). Photograph of head (**A**); thorax (**B**); hindwing (**C**); foreleg (**D**); midleg (**E**); hindleg (**F**); left forewing and hindwing (**G**); left forewing base (**H**); hindwing apex (**I**).

**Figure 3:**
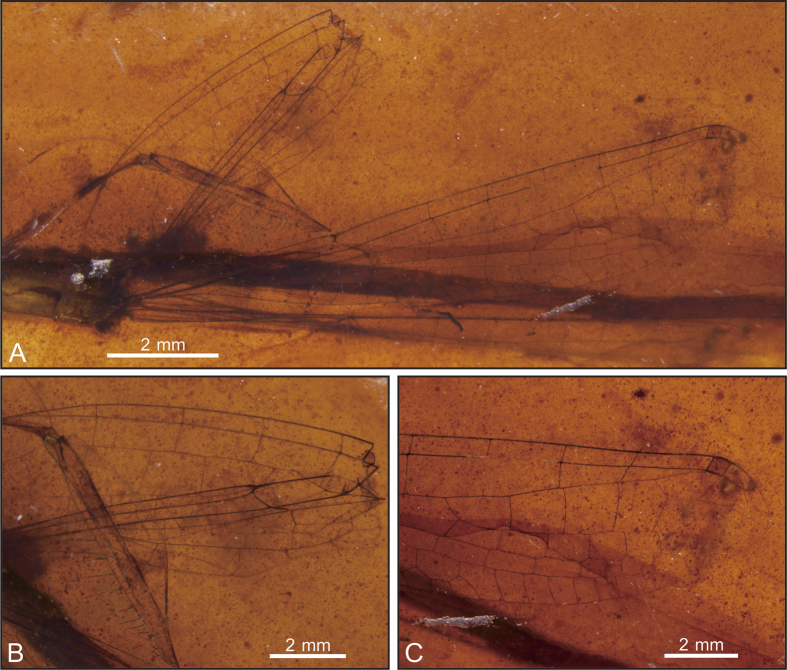
*Yijenplatycnemis huangi* gen. et sp. nov. Paratype (SMNS Bu-137); Photograph of specimen (**A**), right forewing base (**B**) and left forewing apex (**C**).

**Figure 4:**
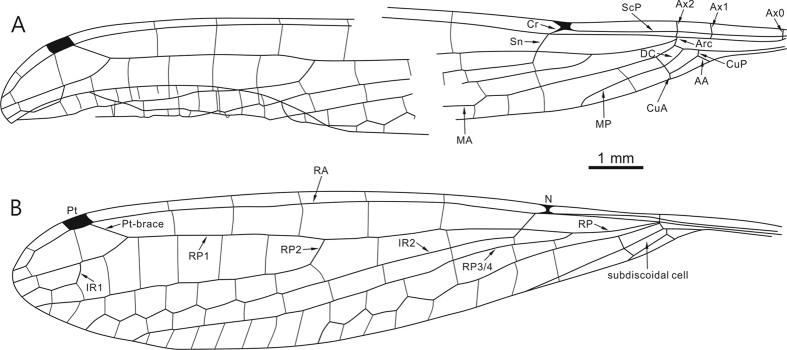
*Yijenplatycnemis huangi* gen. et sp. nov. Paratype (BA16200); line drawing showing venation of left forewing (**A**) and hindwing (**B**) (drawn by DZ). Abbreviations: AA, anterior anal; Arc, arculus; Ax, primary antenodal crossvein; Cr, nodal crossvein; CuA, cubitus anterior; CuP, cubitus posterior; DC, discoidal cell; IR, intercalary radial vein; MA, median anterior; MP, median posterior; N, nodus; Pt, pterostigma; RA, radius anterior; RP, radius posterior; ScP, subcosta posterior; Sn, subnodal crossvein.

**Figure 5:**
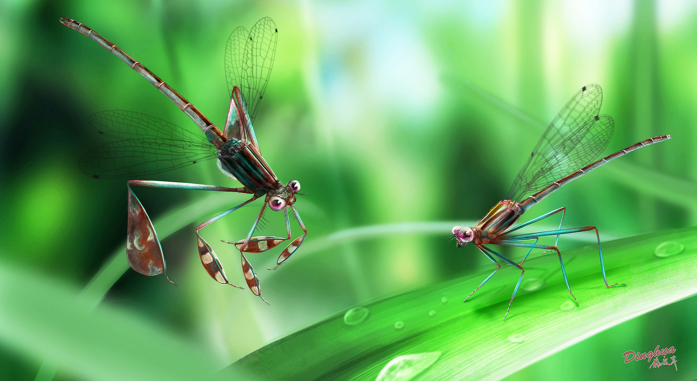
Reconstruction showing the courtship behaviour of *huangi* gen. et sp. nov. from the mid-Cretaceous tropical forest in Burma (drawn by DZ).

(Figures 1, 2, 3, 4, 5).

#### Etymology

The generic name is after Mr Huang Yijen, the donator of the type specimen, and the typical genus *Platycnemis*. The specific name is after Mr. Huang Yijen. Gender masculine.

#### Holotype

NIGP164757, head, thorax and abdomen base well preserved, forewing bases attached to thorax, one fragmentary hindwing near legs, all legs except for right hindleg well preserved; deposited in NIGPAS.

#### Paratype

BA16200, head missing, thorax and abdominal basal segments present, left forewing and hindwing complete, right wing bases badly preserved, only forelegs and midlegs present; temporarily housed at NIGPAS and will eventually be deposited in the Lingpoge Amber Museum in Shanghai. SMNS Bu-137, two forewings well preserved and attached to thorax, a fragmentary leg present; housed at State Museum of Natural History in Stuttgart (Germany).

#### Locality and Horizon

Hukawng Valley, Kachin Province, Myanmar; lowermost Cenomanian, lowermost Upper Cretaceous.

#### Diagnosis

Very small damselfly, complete wing length about 11–14 mm; DC closed and quadrangular with MAb perpendicular to MAa; five postnodal and five postsubnodal crossveins present, somewhat aligned; only one postnodal crossvein present distal of Pt; midfork slightly basal of N; RP1 with strong angle below very long pterostigmal brace; area between RA and RP1 greatly widened distal of Pt; IR2 aligned with Sn; IR1 short, originating below Pt; MA long, ending on posterior wing margin below base of RP2; MP short, one or two cells long; CuA reduced to oblique vein; Pt very small, less than half length of surrounding cells; all tibiae spectacularly expanded, covered with two brown bands, in pod-like sclerite except on metatibiae where of semi-circular shape.

#### Description

Specimen NIGP164757 (Figs 1A and 2A–F), body well preserved. Head dark (Fig. 2A), 2.74 mm long and 0.97 mm wide; eyes 0.89 mm wide, well separated by gap of 0.79 mm; ocelli located low between eyes; antenna three segmented, with segment 1 short and stout, segment 2 stout and 0.75 mm long, segment 3 slim and 0.76 mm long. Legs well developed, profemur 4.11 mm long and armed with long spines in basal part, protibia 2.66 mm long and 0.61 mm wide (Fig. 2D), tarsus 0.62 mm long (claws excluded); mesofemur 5.03 mm long, mesotibia 3.06 mm long and maximum 0.81 mm wide, tarsus 0.74 mm long (Fig. 2E); metafemur 9.02 mm long, metatibia 6.63 mm long and maximum 2.96 mm wide, tarsus 0.91 mm long (Fig. 2F); paired long spines present on tibia and tarsi; tibia armed with about ten pairs of spines; tarsi slightly curved, three segmented, with length of third tarsomere equal to first two tarsomeres combined; basal tarsomere armed with two pairs of long spines, second and third tarsomere armed with four pairs of spines; apical claws symmetrical, 0.12–0.16 mm long.

Specimen BA16200 (Figs 1C–D and 2G–I), left hindwing complete. Wing length 14.07 mm, width at level of N 1.42 mm; length from wing base to Arc 2.24 mm, from Arc to N 2.03 mm, from N to Pt 8.33 mm, from Pt to wing apex 1.45 mm. Primary antenodal crossveins present (Fig. 2H), Ax0 close to wing base, Ax1 1.29 mm distal of Ax0, Ax2 0.64 mm distal of Ax1; no secondary antenodal and antesubnodal crossveins present. Five postnodal and five postsubnodal crossveins present before Pt, somewhat aligned. One postnodal and one postsubnodal crossvein present distal of Pt, non-aligned (Fig. 2I). Arc angular and aligned with Ax2. DC basally closed, free and rectangular, 0.8 mm long and 0.18 mm wide. Subdiscoidal cell free and elongate, 0.81 mm long and 0.2 mm wide. Nodal structures well preserved, Sn aligned with Cr. Midfork (base of RP3/4) slightly basal of N; RP3/4 curved, reaching posterior wing margin just below base of Pt brace. Base of IR2 aligned with Sn, one cell and 0.93 mm distal of midfork; IR2 basally straight but distally zigzagged, ending on posterior wing margin just below Pt. RP2 originating three cells distal of Sn, equidistant between N and Pt, lying 3.6 mm distal of Sn. IR1 originating below end of Pt and six cells distal of base of RP2. RP1 with strong angle below pterostigmal brace. MA basally slightly curved but distally zigzagged, reaching posterior wing margin just below base of RP2. MP curved and short, covering two cells. CuA short, reduced to oblique vein. Pt quite small, rectangular and hyaline, 0.47 mm long and 0.22 mm wide.

Specimen SMNS Bu-137 (Fig. 3) shares all wing characters of specimen BA16200 (Fig. 4) besides following differences: wing short and 11.5 mm long; MP one or two cells long; IR1 originated below Pt base, and five cells distal base of RP2.

#### Remarks

*Y. huangi* has a short vein IR1 originating below the distal side of the pterostigma which is only present in a few damselflies, viz. the platycnemidid *Palaeodisparoneura burmanica* Poinar, Bechly and Buckley, 2010, the hemiphlebiid *Burmahemiphlebia zhangi* Zheng *et al*.^27^, the dysagrionid *Burmadysagrion zhangi* Zheng, Wang and Nel, 2016, and the recent perilestid genus *Perilestes* Hagen in Selys-Longchamps, 1862. *Perilestes* species have the base of RP3/4 and IR2 distal of the nodus, differentiating them from *Y. huangi*^29^. *Burmadysagrion zhangi* has a unique discoidal cell (anterior and posterior sides not parallel, and the basal side longer than the distal side), long MP and CuA, star-shaped Pt, and can thus be easily distinguished from *Y. huangi*^30^. *Y. huangi* has a rectangular discoidal cell, short CuA, RP1 with strong angle below Pt base as in both *P. burmanica* and *Burmahemiphlebia zhangi*^23^,^30^, but differs from the latter two in having only one postnodal crossvein distal of Pt, a smaller Pt, and expanded male tibiae. However, *Burmahemiphlebia zhangi* has a basally open discoidal cell in the forewing, closed in the hindwing, quite different from *Y. huangi*, while *P. burmanica* has a closed discoidal cell more like *Y. huangi*.

Very few modern male damselflies have expanded tibia, namely *Platycypha* Fraser, 1949 (Chlorocyphidae), *Platycnemis* Burmeister, 1839, *Proplatycnemis* Kennedy, 1920, *Copera* Kirby, 1890, *Matticnemis* Dijkstra, 2013 (tibiae weakly expanded) and *Pseudocopera* Fraser, 1922 (Platycnemidae: Platycnemidinae)^32^. Affinities of *Y. huangi* with Chlorocyphidae are excluded because of the different wing venation (numerous antenodals, long CuA, no angular RP1 in Chlorocyphidae)^33^.

The wing venation of *Y. huangi* is very similar to that of the fossil platycnemid *P. burmanica*. Platycnemididae, called white-legged damselflies, currently consist of over 400 species, widely distributed in the Old World^34^,^35^,^36^. They are currently divided into six modern subfamilies^35^ plus the Cretaceous Palaeodisparoneurinae Poinar *et al*.^9^. Despite the fact that the type specimen of *P. burmanica* is a male without expanded tibiae, *Y. huangi* has all the characters listed in the diagnosis of this subfamily^23^. On the other hand, *Y. huangi* strongly differs from modern Platycnemidinae in the very short veins IR1, MP and CuA (apomorphies of Palaeodisparoneurinae), and the broad area between RP1 and IR2. Here we establish a new genus for *Y. huangi* and place it in the Palaeodisparoneurinae. This attribution implies a convergent evolution in the expanded tibiae between *Y. huangi* and modern Platycnemidinae.

## Discussion

Expanded legs are not common in male insects and are normally used for courtship. Some extant male nomiine bees have expanded tibiae using for clasping the females in case of separation during courtship^37^,^38^. Within recent damselflies, male *Platycypha* and *Platycnemis* species have expanded tibiae used for courtship displays^39^. *Platycypha* has all six tibiae expanded, but not as much as *Y. huangi*. Male *P. lacustris* Förster, 1914 has the most expanded tibia within *Platycypha* species, with the outer dilation of hind tibiae 3–5 times wider than the shaft^40^,^41^. This small size does not approach *Y. huangi*. Also, *Platycypha* normally has a different coloration of the inner and outer sides of the tibiae for different functions^42^, unlike *Y. huangi* which has hyaline tibiae and similar pigmented colour on both sides. Male *Platycnemis* are characterized by feather-like tibiae and wide mid and hind tibiae. Besides the Japanese endemic *P. echigoana* Asahina, 1955, all *Platycnemis* species have more or less broadened, flattened and symmetrical tibiae in the middle and hind legs^43^,^44^. *Platycnemis phasmovolans* Hämäläinen 2003 has the most expanded tibiae among modern Platycnemididae, 5.5 mm long and 2 mm broad at the widest point in the hindleg^44^. However, this tibial size is smaller than that of *Y. huangi*, which is 6.6 mm long and 3 mm wide. Unlike *Platycypha* and *Platycnemis*, the tibiae of *Y. huangi* are hyaline, partly covered with two narrow brown bands, and asymmetric with a pod-like shape, especially the hind tibiae which are semi-circular in shape. However, male *Platycnemis* have more expanded tibiae, suggesting closer similarities in the sexual behaviour with *Y. huangi*.

During courtship, male *Platycypha caligata* Selys-Longchamps 1853 waves the white anterior surface of all six laterally enlarged tibiae at the females, but uses the posterior surface of the tibiae for intra-sexual signaling during territorial defence^42^,^45^,^46^,^47^,^48^. Similarly, male East Asian *Platycnemis* species with expanded, feather-like tibiae^49^ well differentiated from the females, exhibit a strong sexual dimorphism^50^. The males display their white legs in a fluttering flight in front of females before mating^39^,^44^,^50^. By morphological inference, the six extremely expanded tibiae of *Y. huangi* could also have a signaling function for courtship displays. *Platycypha* has all six tibiae expanded, but all less so than *Y. huangi* in size. *Platycnemis* has more expanded mid and hind tibiae, but is still smaller than *Y. huangi*. These more expanded fossil tibiae suggest an extreme adaptation for courtship behaviour. More importantly, unlike *Platycypha* and *Platycnemis*, the tibiae of *Y. huangi* are asymmetric and pod-shaped, especially the hindleg tibia with a semi-circular outline. This pod-like shape would make waving slower due to air resistance. *Y. huangi* waving its giant pod-like tibiae would make males more easily noticed and attract female attention (Fig. 5), increasing mating opportunities and implying sexual selection.

The tibial shape of *Y. huangi* also resembles the wings of some members of the extinct neuropteran families Kalligrammatidae and Saucrosmylidae (lacewings). The hindleg tibia of *Y. huangi* have a semi-circular shape, almost the same as the hindwings of the Jurassic saucrosmylid *Daohugosmylus castus* Liu *et al*.^51^. The fore and mid tibiae are also like the wings of some Cretaceous lacewings of Palaeoleontidae Martins-Neto, 1992^52^,^53^. Furthermore, the tibiae of *Y. huangi* are hyaline and partly covered with two narrow brown bands, making them even more like pigmented wings. In addition, there is an eye-shaped spot in the middle of the hindleg, quite like the wing spots in Kalligrammatidae and some recent butterfly eyespots. These well-developed eyespots were and are used to make a conspicuous and contrasting display to intimidate vertebrate predators or protect the body by deflecting an attack to the wings^54^,^55^,^56^. Deflective eyespots in butterflies and fossil lacewings are smaller than deimatic ones and both are never on the legs, but dragonflies are predators with good eyesight, and the tiny ones in *Y. huangi* may have less to do with paralleling fossil lacewings in deflecting nearby predators and more to do with raising the interest of females (cf. peacock eyespots^57^). Some recent damselflies, such as male *Calopteryx hamorrhoidalis*, with higher wing pigmentation, are more likely to defend their territories and obtain more matings^58^. That none of the pigmented tibiae in *Y. huangi* are damaged, however, suggests they did not precipitate an aggressive response.

Our new fossil indicates that the *Platycnemis*-type of courtship behaviour originated at least 100 million years ago. The exaggerated tibiae probably also made them fly slowly. They probably found it less easy to escape from new predators (small birds more efficient than pterosaurs), thus adding more risk in their fancy flight.

## Methods

Photographs were taken using a Zeiss Stereo Discovery V16 microscope system with Zen software. In most instances, incident and transmitted light were used simultaneously. All images are digitally stacked photomicrographic composites of approximately 40 individual focal planes obtained using the free software Combine ZP for a better illustration of the 3D structures. The line drawings were prepared from photographs using image-editing software (CorelDraw X7 and Adobe Photoshop CS6). Specimen NIGP164757 is housed at the Nanjing Institute of Geology and Palaeontology, Chinese Academy of Sciences (NIGPAS). Specimen BA16200 is currently in NIGAPS but will be finally deposited in the Lingpoge Amber Museum in Shanghai (China). Specimen SMNS Bu-137 is housed at State Museum of Natural History in Stuttgart (Germany).

The nomenclature of the dragonfly wing venation used in this paper is based on the interpretations of Riek^59^ and Riek & Kukalová-Peck^60^, as modified by Nel *et al*.^61^ and Bechly^62^. The higher classification of fossil and extant Odonatoptera, as well as family and generic characters followed in the present work, are based on the phylogenetic system proposed by Bechly^62^ and Dijkstra *et al*.^63^ for the phylogeny of extant Zygoptera.

## Additional Information

**How to cite this article:** Zheng, D. *et al*. Extreme adaptations for probable visual courtship behaviour in a Cretaceous dancing damselfly. *Sci. Rep.* **7**, 44932; doi: 10.1038/srep44932 (2017).

**Publisher's note:** Springer Nature remains neutral with regard to jurisdictional claims in published maps and institutional affiliations.

## References

[b1] Eberhard W. G. Copulatory courtship and cryptic female choice in insects. Biol. Rev. 66, 1–31 (1991).

[b2] Eberhard W. G. Evidence for widespread courtship during copulation in 131 species of insects and spiders, and implications for cryptic female choice. Evolution 48, 711–733 (1994). 10.1111/j.1558-5646.1994.tb01356.x 28568272

[b3] Waage J. K. Female and male interactions during courtship in *Calopteryx maculata* and *Calopteryx dimidiata* (Odonata: Calopterygidae): Influence of oviposition behaviour. Anim. Behav. 32, 400–404 (1984).

[b4] Cordero Rivera A. & Andrés J. A. Male coercion and convenience polyandry in a Calopterygid damselfly (Odonata). J. Insect Sci. 2, 1–7 (2002). 15455048 10.1093/jis/2.1.14 PMC355914

[b5] Hilton D. F. J. Reproductive behaviour of *Leucorrhinia hudsonica* (Selys) (Odonata: Libellulidae). J. Kansas Entomol. Soc. 57, 580–590 (1984).

[b6] Tsubaki Y., Samejima Y. & Siva-Jothy M. T. Damselfly females prefer hot males: higher courtship success in males in sunspots. Behav. Ecol. Sociobiol. 64, 1547–1554 (2010).

[b7] Isles T. E. The socio-sexual behaviour of extant archosaurs: implications for understanding dinosaur behaviour. Hist. Biol. 21, 139–214 (2009).

[b8] Boucot A. J. & Babcock L. E. Evolutionary paleobiology of behavior and coevolution 1–705 (Elsevier Science Press, 1990).

[b9] Boucot A. J. & Poinar G. Jr. Fossil behavior compendium 147–152 (CRC Press, 2011).

[b10] Li S., Shih C., Wang C., Pang H. & Ren D. Forever love: the hitherto earliest record of copulating insects from the Middle Jurassic of China. PLoS ONE 8, e78188 (2013). 24223138 10.1371/journal.pone.0078188 PMC3819342

[b11] Peñalver E. . Thrips pollination of Mesozoic gymnosperms. PNAS 109, 8623–8628 (2012). 22615414 10.1073/pnas.1120499109 PMC3365147

[b12] Cai C. . Early origin of parental care in Mesozoic carrion beetles. PNAS 111, 14170–14174 (2014). 25225362 10.1073/pnas.1412280111 PMC4191754

[b13] Wang B. . Brood care in a 100-million-year-old scale insect. eLife 4, e05447 (2015). 10.7554/eLife.05447 PMC4378507 25824055

[b14] Wang Q., Shih C. & Ren D. The earliest case of extreme sexual display with exaggerated male organs by two Middle Jurassic mecopterans. PLoS ONE 8, e71378 (2013). 23977031 10.1371/journal.pone.0071378 PMC3743757

[b15] Rasnitsyn A. P. New Hymenoptera from the Jurassic and Cretaceous of Asia. Paleontol. Zhurn. 3, 98–108 (1977).

[b16] Zhang Q., Zhang H., Rasnitsyn A. P., Wang H. & Ding M. New Ephialtitidae (Insecta: Hymenoptera) from the Jurassic Daohugou Beds of Inner Mongolia, China. Palaeoworld 23, 276–284 (2014).

[b17] Huang D., Nel A., Cai C., Lin Q. & Engel M. S. Amphibious flies and paedomorphism in the Jurassic period. Nature 495, 94–97 (2013). 23426262 10.1038/nature11898

[b18] Wappler T., Guilbert E., Labandeira C. C., Hörnschemeyer T. & Wedmann S. Morphological and Behavioral Convergence in Extinct and Extant Bugs: The Systematics and Biology of a New Unusual Fossil Lace Bug from the Eocene. PLoS ONE 10, e0133330 (2015). 26267108 10.1371/journal.pone.0133330 PMC4534043

[b19] Bechly G., Brauckmann C., Zessin W. & Gröning E. New results concerning the morphology of the most ancient dragonflies (Insecta: Odonatoptera) from the Namurian of Hagen-Vorhalle (Germany). J. Zool. Sys. Evol. Research 39, 209–226 (2001).

[b20] Kania I., Wang B. & Szwedo J. *Dicranoptycha* Osten Sacken, 1860 (Diptera, Limoniidae) from the earliest Upper Cretaceous Burmese amber. Cretaceous Res. 52, 522–530 (2015).

[b21] Shi G. . Age constraint on Burmese amber based on U-Pb dating of zircons. Cretaceous Res. 37, 155–163 (2012).

[b22] Cruickshank R. D. & Ko K. Geology of an amber locality in the Hukawng Valley, northern Myanmar. J. Asian Earth Sci. 21, 441–455 (2003).

[b23] Poinar G. Jr, Bechly G. & Buckley R. First record of Odonata and a new subfamily of damselflies from Early Cretaceous Burmese amber. Palaeodiversity 3, 15–22 (2010).

[b24] Bechly G. & Poinar G. Jr. *Burmaphlebia reifi* gen. et sp. nov., the first anisozygopteran damsel-dragonfly (Odonata: Epiophlebioptera: Burmaphlebiidae fam. nov.) from Early Cretaceous Burmese amber. Hist. Biol. 25, 233–237 (2013).

[b25] Huang D., Azar D., Cai C. & Nel A. New damselfly genera in Cretaceous Burmese amber attributable to the Platystictidae and Platycnemididae Disparoneurinae (Odonata: Zygoptera). Cretaceous Res. 56, 237–243 (2015).

[b26] Schädel M. & Bechly G. First record of Anisoptera (Insecta: Odonata) from mid-Cretaceous Burmese Amber. Zootaxa 4103, 537–549 (2016). 27394756 10.11646/zootaxa.4103.6.4

[b27] Zheng D. . New damselflies (Odonata: Zygoptera: Hemiphlebiidae, Dysagrionidae) from mid-Cretaceous Burmese amber. Alcheringa doi: 10.1080/03115518.2016.1164402 (2016).

[b28] Zheng D., Zhang Q., Chang S.-C. & Wang B. A new damselfly (Odonata: Zygoptera: Platystictidae) from mid-Cretaceous Burmese amber. Cretaceous Res. 63, 142–147 (2016).

[b29] Zheng D., Wang B., Jarzembowski E. A., Chang S.-C. & Nel A. The first fossil Perilestidae (Odonata: Zygoptera) from mid-Cretaceous Burmese amber. Cretaceous Res. 65, 199–205 (2016).

[b30] Zheng D., Wang B., Jarzembowski E. A., Chang S.-C. & Nel A. Burmadysagrioninae, a new subfamily (Odonata: Zygoptera: Dysagrionidae) from mid-Cretaceous Burmese amber. Cretaceous Res. 67, 126–132 (2016).

[b31] Zheng D., Jarzembowski E. A., Chang S.-C. & Wang B. A new true dragonfly (Odonata, Anisoptera, Gomphaeschnaoidini) from mid-Cretaceous Burmese amber. P. Geologist Assoc. doi: 10.1016/j.pgeola.2016.07.006 (2016).

[b32] Dijkstra K.-D. B. Three new genera of damselflies (Odonata: Chlorocyphidae, Platycnemididae). Int. J. Odonatol. 16, 269–274 (2013).

[b33] Fraser F. C. A revision of the Chlorocyphidae with notes on the classification of the *Selysia* species *rubida, glauca, cyanifrons* and *curta*. Bull. Inst. Roy. Sci. Nat., Belg. 25, 1–50 (1949).

[b34] Orr A. G. & Kalkman V. J. *Arrhenocnemis parvibullis* sp. nov. (Odonata: Platycnemididae), a new calicnemiine damselfly from Papua New Guinea, with a description of the female of *A. amphidactylis* Lieftinck, 1949. Aust. Entomol. 37, 137–146 (2010).

[b35] Dijkstra K.-D. B., Kalkman V. J., Dow R. A., Stokvis F. R. & van Tol J. Redefining the damselfly families: a comprehensive molecular phylogeny of Zygoptera (Odonata). Syst. Entomol. 39, 68–96 (2014).

[b36] Theischinger G., Gassmann D. & Richards S. J. *Macrocnemis gracilis*, a new genus and species of Idiocnemidinae (Zygoptera: Platycnemididae) from Papua New Guinea. Zootaxa 3990, 429–437 (2015). 26250243 10.11646/zootaxa.3990.3.7

[b37] Wcislo W. T. & Buchmann S. L. Mating behaviour in the bees, *Dieunomia heteropoda* and *Nomia tetrazonata*, with a review of courtship in Nomiinae (Hymenoptera: Halictidae). J. Nat. Hist. 29, 1015–1027 (1995).

[b38] Wcislo W., Minckley R. & Spangler H. Pre-copulatory courtship behaviour in a solitary bee, *Nomia triangulifera* Vachal (Hymenoptera: Halictidae). Apidologie 23, 431–442 (1992).

[b39] Preston-Mafham R. & Preston-Mafham K. The encyclopaedia of land invertebrate behaviour 1–320 (Blamford, London, 1993).

[b40] Förster F. Beiträge zu den Gattungen und Arten der Libellen. Arch. Naturgesch. (A) 80, 59–83 (1914).

[b41] Dijkstra K.-D. B. The systematist’s muse—two new damselfly species from ‘Elisabetha’ in the Congo Basin (Odonata: Chlorocyphidae, Platycnemididae). Zool. Med. Leiden 82, 15–27 (2008).

[b42] Jennions M. D. Tibial coloration, fluctuating asymmetry and female choice behaviour in the damselfly *Platycypha caligata*. Anim. Behav. 55, 1517–1528 (1998). 9641997 10.1006/anbe.1997.0656

[b43] Asahina S. A new platycnemidid damselfly from Japan. Akitu 4, 101–104 (1955).

[b44] Hämäläinen M. *PIatycnemis phasmovolans* sp. nov.—an extraordinary damselfly from Laos with notes on its East Asian congeners (Odonata: Platycnemididae). TOMBO, Matsumoto 46, 1–7 (2003).

[b45] Selys-Longchamps E. de. Synopsis des Caloptérygines. Bull. Acad. Roy. Belg. 20, 1–73 (1853).

[b46] Robertson H. M. Mating behaviour and its relationship to territoriality in *Platycypha caligata* (Selys) (Odonata: Chlorocyphidae). Behaviour 74, 11–26 (1982).

[b47] Robertson H. M. Courtship displays and mating behaviour of three species of Chlorocyphidae (Zygoptera). Odonatologica 110, 53–58 (1982).

[b48] Telford S. R., Barnett M. & Polakow D. A. The functional significance of tibial displays in the damselfly *Platycypha caligata* (Selys) (Odonata: Chlorocyphidae). J. Insect Behav. 9, 835–839 (1996).

[b49] Dijkstra K.-D. B. & Kalkman V. J. Phylogeny, classification and taxonomy of European dragonflies and damselflies (Odonata): a review. Org. Divers. Evol. 12, 209–227 (2012).

[b50] Battin T. Geographic variation analysis among populations: the case of *Platycnemis pennipes* (Pallas, 1771) (Insecta: Odonata: Zygoptera) in the Aegean. J. Biogeogr. 19, 391–400 (1992).

[b51] Liu Q. . A new saucrosmylid lacewing (Insecta, Neuroptera) from the Middle Jurassic of Daohugou, Inner Mongolia, China. Alcheringa 38, 301–304 (2014).

[b52] Heads S. W., Martill D. M. & Loveridge R. F. An exceptionally preserved antlion (Insecta, Neuroptera) with colour pattern preservation from the Cretaceous of Brazil. Palaeontology 48, 1409–1417 (2005).

[b53] Martins-Neto R. G. Neurópteros (Insecta, Planipennia) da Formação Santana (Cretáceo Inferior), Bacia do Araripe, Nordeste do Brasil. V. Aspectos filogenéticos, paleoecológicos, paleobiogeográficos e descrição de novos taxa. An. Acad. Bras. Ciênc. 64, 117–148 (1992).

[b54] Vallin A., Jakobsson S., Lind J. & Wiklund C. Prey survival by predator intimidation: an experimental study of peacock butterfly defense against blue tits. Proc. R. Soc. B 272, 1203–1207 (2005). 10.1098/rspb.2004.3034 PMC1564111 16024383

[b55] Prudic K. L., Stoehr A. M., Wasik B. R. & Monteiro A. Eyespots deflect predator attack increasing fitness and promoting the evolution of phenotypic plasticity. Proc. R. Soc. B 282, 20141531 (2014). 10.1098/rspb.2014.1531 PMC4262162 25392465

[b56] Labandeira C. C. . The evolutionary convergence of mid-Mesozoic lacewings and Cenozoic butterflies. Proc. R. Soc. B 283, 20152893 (2016). 10.1098/rspb.2015.2893 PMC4760178 26842570

[b57] Petrie M. & Tim H. & Carolyn, S. Peahens prefer peacocks with elaborate trains. Anim. Behav. 41, 323–31 (1991).

[b58] Córdoba-Aguilar A. Wing pigmentation in territorial male damselflies, *Calopteryx haemorrhoidalis*: a possible relation to sexual selection. Anim. Behav. 63, 759–766 (2002).

[b59] Riek E. F. A new collection of insects from the Upper Triassic of South Africa. Ann. Natal Mus. 22, 791–820 (1976).

[b60] Riek E. F. & Kukalová-Peck J. A new interpretation of dragonfly wing venation based upon early Carboniferous fossils from Argentina (Insecta: Odonatoidea) and basic characters states in pterygote wings. Can. J. Zool. 62, 1150–1166 (1984).

[b61] Nel A., Martínez-Delclòs X., Paicheler J. C. & Henrotay M. Les ‘Anisozygoptera’ fossiles. Phylogénie et classification (Odonata). Martinia Num. Hors Sér. 3, 1–311 (1993).

[b62] Bechly G. Morphologische Untersuchungen am Flügelgeäder der rezenten Libellen und deren Sta mmgruppenvertreter (Insecta; Pterygota; Odonata), unter besonderer Berücksichtigung der Phylogenetischen Systematik und des Grundplanes der Odonata. Petalura 2, 1–402 (1996).

[b63] Dijkstra K.-D. B., Kalkman V. J., Dow R. A., Stokvis F. R. & van Tol J. Redefining the damselfly families: a comprehensive molecular phylogeny of Zygoptera (Odonata). Syst. Entomol. 39, 68–96 (2014).

